# Supplementary material for: Are carbon emissions trading and green financial instruments synergistic? -Comprehensive quantitative research based on content analysis
Source: PLoS One. 2024 Mar 7;19(3):e0298601. doi: 10.1371/journal.pone.0298601 (PMC10919601; doi:10.1371/journal.pone.0298601)
Supplement: S1 Data — (ZIP) [file pone.0298601.s001.zip › Data/地级市Z_F工作报告.zip!/║┌┴·╜¡/╣■╢√▒⌡╩╨/└┤╫╘╣1⁄2╓┌║┼í╛┬φ┐╦╩2╛▌═°í┐_2004.docx]

各位代表：

　　现在，我代表市人民政府向大会作工作报告，请予审议，并请列席会议的政协委员提出意见。

　　一、2003年工作回顾

　　2003年是我们面临重大机遇，经受严峻考验，取得显著成绩的一年。一年来，我们高举邓小平理论和“三个代表”重要思想伟大旗帜，贯彻党的十六大和十六届三中全会精神，落实省委“努力快发展，全面建小康”的总体要求，以振兴老工业基地为契机，深化改革，调整结构，推进“五个基地、一个中心、一座名城”建设，取得了全面建设小康社会的良好开局。实现哈尔滨市生产总值1414.8亿元，增长13.5%，为1997年以来最高水平。财政一般预算收入135.3亿元，增长14.8%，其中地方财政一般预算收入76.4亿元，增长16.2%。完成固定资产投资436亿元，增长20.7%。社会消费品零售总额624.2亿元，增长11.6%。进出口贸易总值19.1亿美元，增长11.3%。城市居民人均可支配收入7892.7元，增长12.7%；农民人均纯收入2943.2元，增长6%。

　　在经济形势复杂多变，多种自然灾害频发，非典疫情突然袭来的严峻考验下，我市经济社会事业仍然快速发展，是我们坚持用“三个代表” 重要思想指导实践、解决问题、推动工作的结果。

　　（一）坚持先进生产力的发展要求，加快了经济发展步伐

　　抢抓机遇，深化改革，加快二、三产业发展。全面启动老工业基地调整改造，提出建立新体制、引入新机制、构筑新结构、采用新方式、注入新活力的“五新”目标，制定了《关于加快推进市属国有工业企业改革改制工作的意见》，规划了590个项目。在国家重点支持的第一批100个国债项目中，我市列入17个，总投资26.7亿元。争取到国家开发银行政府信用贷款额度170亿元。在国有企业改革中，采用一企一策，开放式改革改制等办法，完成100户国有企业产权制度改革。工业经济实现速度、效益双高增长，实现工业增加值421.3亿元，增长17.7%，经济效益综合指数首次突破100，达到104.2。第三产业快速增长，实现增加值673.9亿元，增长12.7%。旅游业虽然受非典疫情冲击，仍实现收入86.7亿元，增长14.1%，成为全国旅游业的亮点。商业企业改革取得实质性进展，50%的国有商业企业进入产权改革程序，部分企业完成整体转让。引入先进商业业态，多层次、开放式、竞争型格局正在形成。现代物流业发展较快，启动城市物流园区建设，7家国内外较大物流企业落户哈东物流作业基地。金融、交通、房地产、会展等服务业稳步发展。

　　以振兴老工业基地为契机，大力吸纳外资和民间资本。加大专业招商力度，实际利用外资2.7亿美元，增长10.1%。开发区带动作用明显，实际利用外资1.4亿美元，占全市的51.7%。加强横向联合，跨地区资金到位39.1亿元，增长16.6%。民营经济发展提速，实现增加值797.2亿元，占全市GDP的56.3%。民间投资203亿元，占全市投资的46.6%。

　　促进城乡经济协调发展，努力增加县（市）财政和农民收入。坚持“统筹城乡经济社会发展”方针，按照县（市）财政和农民“双增收”目标，加快推进农区工业化、农村城镇化和农业产业化，县域经济出现较大转机，12县（市）生产总值和财政收入增长幅度，首次超过全市平均水平，成为全市国民经济的亮点。实施结构调整，农业在多灾之年减产增收，实现农林牧渔业总产值351.1亿元，增长6.6%，农民人均纯收入提高166.6元。推进种植业与畜牧业“主辅换位”，畜牧业实现产值155.5亿元，占农林牧渔业总产值的44.3%。绿色食品种植面积180万亩，增长20%。高油、高蛋白大豆等优质农作物面积1836万亩，优质率提高4个百分点。龙头企业进一步壮大，产业化经营带动作用明显增强。与省农垦总局开展了横向联合。农村工业园区和小城镇建设取得新进展，农村劳动力转移步伐加快。深化农村改革，在税费改革基础上，全部取消农林特产税，减轻农民负担目标完成率100%。

　　依靠科技创新，推动经济增长方式转变。高新技术产业实现产值400亿元，增长33%。民营科技企业技工贸总收入245亿元，增长22.5%。推进知识创新和技术创新，取得重大科技成果956项，获国家专利880项。以企业为主体的技术创新体系建设步伐加快，重大高新技术发展项目进展顺利。加大技术改造投入，完成工业投资98.1亿元，企业技术水平进一步提升。推进企业信息化建设，试点企业达到60户。加大民营科技企业扶持力度，“小巨人”企业不断壮大。

　　转变政府职能，着力改善经济发展环境。推进政务公开，出台了《哈尔滨市政务公开规定》等6项政府规章。清理行政审批事项，减少和转变行政管理事项230项，市行政服务大厅建设顺利进行。转变工作作风，创新工作方式，采用“课题制”方法，围绕事关经济社会发展的重点领域、关键环节抓落实，围绕难点、热点问题抓落实，围绕实现广大人民群众的切身利益抓落实，提高了政府工作效率。

　　（二）坚持先进文化的前进方向，促进了社会事业不断进步

　　坚持以人为本，推进城市管理升级。城市发展，三分建设七分管理。管理的过程，实质就是向城市注入“文化”的过程。在政府工作中，加强制度建设，逐步形成城市发展的总体理念和原则。坚持改革创新。在实施振兴老工业基地战略方面，提出“五新”思路；在推动县域经济发展方面，提出县（市）财政和农民“双增收”的目标，把农业和农村工作放在县域经济大格局中整体推进；在城市建设方面，提出由建设“水泥城市”向建设生态型园林城市转变，由单纯城市功能建设向功能建设与文化延展并重转变，实行市区联动、区企联动、工作重心下移的推进机制；在实施开发区牵动战略方面，实行一区带多园，实现一个国家级开发区带动多个园区发展。坚持推动科技进步。科技对经济社会发展的促进作用增强，数字哈尔滨和网络型政府建设成效显著。坚持市场化原则。对城市供排水、供热、清冰雪等公益事业进行改革，组建了供排水集团、物业供热集团，把市场机制引入政府公共服务领域。加大经营城市力度，由过去单个项目经营向建立经营机制转变，组建城市建设投资公司，城市建设投融资体制进一步完善。

　　突出文化特色，提高城市建设水平。以建设生态型园林城市为目标，注重延展城市历史文化特色，城市建设实现了质的飞跃。城市建设投资87.5亿元，增长18.9%。完成新一轮城市总体规划和41项控详规划。东直路改造、香坊大街拓宽等15路5桥重点工程全部竣工。植树141万株，城区移植大树1.4万株，成活率98%以上。新增绿地546公顷，城市绿化覆盖率由28%提高到31.7%。改造中央大街二期、果戈里大街、三大动力路、哈药路、高丽一条街等文化街区，城市景观特色和文化品位更加突出。完成太阳岛综合改造一期、北方森林动物园一期工程。磨盘山水库供水工程建设进展顺利，完成一期工程总量的23.4%，文昌污水处理厂一、二期工程投入使用，沿江污水截流及处理、通乡公路等新开工项目进展顺利，向阳生活垃圾无害化处理场工程、道里区集中供热工程全面启动。以会展中心周边地区、沿江风景长廊、铁路沿线和火车站广场环境综合整治为重点，完成综合整治项目500个。治理烟尘、污水排放污染源350个。城市综合执法工作有新进展，整顿城市交通秩序取得初步成果。

　　旅游产业综合开发成果显著。以冰雪文化为核心，注重多元文化融合，塑造特色旅游品牌。第十九届中国·哈尔滨国际冰雪节盛况空前，第四届冰雪大世界、太阳岛雪博会规模和水平创历年之最。建设太阳岛风景名胜区、金源文化旅游开发区等重点项目，放大了旅游文化品牌效应。在做大做强冰雪旅游基础上，积极培育欧陆风光游、生态游、异域风情游等四季旅游热点。社会主义精神文明和民主法制建设进一步加强。贯彻《公民道德建设实施纲要》，深入开展“改陋习、树新风、塑形象”活动，公民道德素质和城市文明程度不断提高。荣获全国双拥模范城“五连冠”称号。文化事业日益繁荣，推出一批有影响力的文艺作品。完成市图书馆扩建工程。加强民主法制建设，自觉接受人大依法监督和政协民主监督，办复人大代表建议268件，政协委员提案887件，办复率100%。提请市人大常委会审议地方性法规草案7件，制定市政府规章17件、规范性文件33件。落实行政执法责任制，依法行政水平进一步提高。深入开展“四五”普法教育，市民法律意识明显增强。加强市场监管，经济秩序进一步好转。切实加强安全工作，各类事故发生次数和伤亡人数有所下降。信访工作得到加强，办理群众来信来访、来电和建议58.6万件次。严厉打击“法轮功”等邪教组织，深入开展“警民打防工程”，刑事案件总量下降29.8%，在全国同类城市中发案率最低。教育、卫生等各项事业快速发展。新建、扩建10所优质高中，改造23所教学设施薄弱学校。城市95%、农村70%的学校实现“校校通”。加强教师队伍培训，教师专业化水平进一步提高。成功举办第十届全国冬季运动会，全民健身运动更加广泛、活跃。医疗卫生条件和预防保健水平进一步提高，5个区急救分中心投入使用，12县（市）疾病控制中心建成4个，完成主体工程6个。社区卫生服务网络进一步完善，爱国卫生运动深入开展。认真贯彻计划生育基本国策，人口自然增长率3.3‰。

　　人防、新闻出版、广播电视、民族宗教、外事侨务、对台、老龄、残联、武装、档案和地方志等方面工作取得新成绩。（三）坚持实现好、维护好、发展好广大人民群众的根本利益，把为民谋利作为工作的出发点和落脚点

　　提高人民生活质量。市区人均住房使用面积15.1平方米，农村居民人均居住面积19.6平方米。拆迁危房棚户区41片，12万居民住房条件得到改善。新辟、调整公交线路17条，更新公交车辆899台。

　　努力解决就业和再就业问题。拓展就业渠道，安置下岗失业人员12.4万人，其中6万名失业人员实现就业，城镇登记失业率3.9%。建立再就业责任体系，落实各项再就业扶持政策，安置下岗职工6.4万人，再就业率达到42.5%。加大职业培训力度，下岗、失业人员就业能力进一步提高。

　　完善社会保障体系。巩固“两个确保”，强化“三条保障线”的衔接。国有企业下岗职工基本生活费、企业离退休人员基本养老金按时足额发放率100%，新增基本养老保险参保企业1640户，企业基本养老保险费征缴率为96%。医疗保险覆盖面扩大，参保人数增加到120万人，失业保险参保人数180万人。完善城市居民最低生活保障配套政策，28万人纳入低保范围，做到应保尽保。应拨付城市居民最低生活保障金及时到位率100%。

　　扶助社会困难群体。推进经常性社会捐助，接收捐助191万元，救助困难群众1.8万人。圆满完成第一轮“一帮一”扶贫解困工程，8328名特困职工全部脱贫。救灾、救济资金及时到位率100%。实施廉租住房、义务教育贫困学生助学金等专项救助办法。加大扶贫开发，20个贫困村、8.2万贫困人口脱贫。解决革命老区300个村（屯）饮水难问题。

　　年初确定为人民群众办好的“10件实事”全部得到落实。

　　各位代表，2003年是令人难忘的一年，突如其来的非典疫情，使全市人民经受了严峻考验。我们按照国家和省的部署，采取积极的应对措施，周密安排，迅速组建领导体系、防治监控体系和应急防疫队伍，依靠全市人民，全力开展防控工作，实现了“力争不发生，确保不扩散”的目标，取得了抗击非典的阶段性胜利，保障了人民群众身体健康和生命安全。在抗击非典斗争中，我们获得了宝贵的精神财富，“万众一心、众志成城，团结互助、和衷共济，迎难而上、敢于胜利”的抗非典精神，将在振兴老工业基地、全面建设小康社会的伟大实践中发扬光大！

　　各位代表，总结过去的工作，可谓“看似寻常最崎岖，成如容易却艰辛”。一年来取得的成绩，是党中央、国务院和省委、省政府及市委正确领导的结果，是市人大、政协、民主党派、工商联和群众团体有力支持和监督的结果，是全市人民团结一致、奋力拼搏的结果。在此，我代表市人民政府向全市各族人民，向驻哈部队和武警官兵，向国家和省驻哈单位，向港澳台同胞、海外侨胞及国际友人，向所有关心、支持、参与哈尔滨建设的同志们、朋友们，致以衷心感谢和崇高敬意!

　　我们清醒地认识到，当前我市经济和社会发展面临的问题还比较突出。国有经济布局战略性调整和产业结构升级滞后，区域经济竞争力提升缓慢；民营经济发展滞后；县域经济基础薄弱，城乡经济统筹发展程度偏低，县级财政和农民收入增长不快；经济外向度低，外贸进出口增长乏力；就业压力加大，社会保障能力较弱；城市管理不尽完善；重特大安全事故没有得到有效控制；政府职能转变不到位，经济发展环境还不够宽松；广大人民群众关注的腐败问题还没有得到有效根治。这些问题，我们将努力加以解决。

　　二、以振兴老工业基地为核心，全力做好2004年各项工作今年将全面实施振兴老工业基地战略，我们面临严峻挑战，除了前面讲到的自身问题外，外部环境也给我们增加了压力：全国各城市竞相发展，我们不进则退，慢进亦退；我国加入世贸组织的过渡期即将结束，我们将更直接面对国际市场竞争。我们也具备许多有利条件：一是世界经济增长加快，我国经济正处在新一轮增长期，特别是制造业和原材料工业发展迅猛，为我们提供了良好的宏观环境。二是国家实施振兴老工业基地战略，将加速国际和国内发达地区资本向东北地区转移。三是我市既是老工业基地，又是粮食主产区，能够更多地享受国家扶持政策。四是我市多年来改革发展的累积效应逐步显现，特别是去年老工业基地调整改造的基础性工作，将在今年显效。五是我市土地资源、产业资源、科技和人力资源相对丰富，电力等能源供应充足。只要正确判断形势，趋利避害，我们有充分的信心，开创振兴老工业基地和全面建设小康社会的新局面。

　　今年政府工作的总体思路是：高举邓小平理论和“三个代表”重要思想伟大旗帜，深入贯彻党的十六大和十六届三中全会精神，按照“努力快发展、全面建小康、振兴哈尔滨”的要求，全面实施振兴老工业基地战略，统筹城乡发展，统筹经济社会发展，扎实开展“招商引资和改善环境年”活动，加快“五个基地、一个中心、一座名城”建设，促进国民经济持续、快速、协调、健康发展和社会全面进步，在全省进一步发挥龙头作用。

　　主要预期目标是：哈尔滨市生产总值增长12%，其中第一、二、三产业分别增长6%、15%、12%；单位GDP能耗、水耗低于全国平均水平；全社会固定资产投资增长15%；地方财政一般预算收入增长9%；社会消费品零售总额增长11.5%；进出口总值增长8%；实际直接利用外资增长10%；城市居民人均可支配收入增长8%，农村居民人均纯收入增长6%以上；城镇登记失业率不超过5.5%；人口自然增长率低于4.9‰。

　　上述目标，是按照振兴老工业基地和全面建设小康社会总体规划制定的，既体现了与时俱进、加快发展的要求，又充分考虑了我市的实际状况。只要我们齐心协力，埋头苦干，就一定能完成既定目标，为实现全面振兴哈尔滨的宏伟蓝图奠定基础。

　　（一）推进国有企业改革，加快产业结构调整

　　今年，老工业基地调整改造以国有企业改革改制为突破口，加大产业整合力度，务求取得阶段性成果，确保工业增加值增长15%，力争达到20%。

　　打好国有企业改革改制攻坚战。一是在产权制度改革方面力求突破。贯彻国家《关于规范国有企业改制工作的意见》，落实我市《关于加快推进市属国有工业企业改革改制工作的意见》，对国有企业进行分类指导，以股份制为重点，采取5种形式，进行脱胎换骨式的改革改造。做大做强一批，对有规模、有实力的国家大企业，以产业基地为依托，壮大配套规模，促进横向并购，推动纵向延伸，形成产业集群；对5户市属优势大企业集团，开展与国内外大公司合资合作，扩大规模和市场占有率，发展成全国同行业优强企业。招商改制一批，对30户有一定优势和发展潜力的企业进行全方位招商，承接国际产业转移项目，吸纳民营资本，实行股份制改造。分块搞活一批，对31户整体改制有困难的企业，实行分立改制。彻底放开一批，对94户中小企业，通过多种方式，改制成混合所有制和民营企业。破产退出一批，对120户劣势企业实施破产关闭，年内争取6户企业列入国家政策性破产计划，符合条件的企业依法破产。搞好国有企业主辅分离，启动12所企办学校分离移交工作。二是在化解国有企业债务方面力求突破。利用国家有关政策，积极与银行、金融资产经营公司衔接，争取折让偿还金融债务或债权回购。严格管理办法和操作程序，防止逃废债务。三是在建立新型劳动关系方面力求突破。按照“积极稳妥、依法有序、因企制宜、分类处理”的原则，全年调整11万名市属国有企业职工劳动关系，力争两年内全部完成24万名市属国有企业职工劳动关系调整任务。

　　以大项目为牵动，做大做强优势产业。对国家已确定支持的17个项目，认真搞好对接，尽快启动实施。完善项目生成机制，围绕国家支持重点，继续生成一批重大项目，争取国家支持。加大技术改造和重组力度，投资100亿元以上，推进机械制造业、高新技术产业、食品和医药工业基地建设。机械制造业投资60亿元，重点实施28个重大项目，实现产值330亿元。高新技术产业以电子信息、生物制药、光机电一体化、新材料、高效节能与环保、高效农业为重点，实现产值530亿元。食品工业重点发展乳制品、啤酒、大豆加工、粮食深加工，做大做强完达山、龙丹、金星、哈啤等龙头企业，实现产值135亿元。医药工业重点发展化学原料药、现代中药系列、滋补保健品等，推进产业重组，放大医药品牌效应，实现产值85亿元。按照政策引导、市场化运作原则，进一步调整工业布局，建设动力园区、汽车园区、医药园区、木材加工园区、高新技术产业园区、对俄科技经贸合作园区。对10户市区内企业实施搬迁改造。

　　完善工作推进机制。成立专门领导机构，负责对上联系、政策衔接和具体工作推进。完善国有资产管理体制，强化国有资产监管机构职能，使国有资产出资人在企业改革改制中真正到位。对市属国有企业，实行一企一策，推进改革改制和资产重组。宣传、推介振兴老工业基地“概念”，实行开放式调整改造。

　　（二）统筹城乡发展，壮大县域经济

　　贯彻落实《中共中央、国务院关于促进农民增加收入若干政策的意见》精神，以增加农民收入为核心，以县（市）财政和农民“双增收”为目标，继续推进农区工业化、农村城镇化、农业产业化，支持呼兰、双城、阿城、尚志、五常5个实力较强县（市）率先发展，鼓励中等县（市）加速崛起，扶持薄弱县奋起追赶。

　　推进农业结构调整。利用国家支持粮食主产区的政策机遇，扶持产粮大县建设国家优质专用粮食基地，发展粮食精深加工，做大做强粮食产业。加快“主辅换位”，继续实施奶业振兴计划，畜牧业产值占农林牧渔业的比重达到46.7%。按照高产、优质、高效、生态、安全的要求，完善农产品标准化、质量安全和认证体系建设。发展优质专用和绿色、无公害农产品，推进玉米、水稻、大豆、肉类、禽蛋、乳品和蔬菜7个产业迅速壮大，绿色、无公害食品监控面积力争达到360万亩，培育一批有机食品基地。继续实施大豆振兴计划，高油、高蛋白大豆生产面积增加到350万亩。扩大饲草、饲料生产面积，优化粮经饲三元结构。以棚室蔬菜和花卉为重点，发展城郊型农业。加强农业基础设施建设，推进农田水利、小流域综合治理和县乡村道路建设，通乡高等级公路实现通车500公里，开工建设哈肇公路呼兰至通河段工程。继续做好退耕还林、还湿、还草工作。搞好农业综合开发和扶贫解困工作，继续实施革命老区300个自然村（屯）饮水工程，入户率达到80%。加强与农垦系统合作，整合农业资源，实现优势互补和经济互动。

　　加快发展县域工业。以振兴老工业基地为契机，引导县域企业加入城市工业分工协作体系，发展配套产品。推进工业园区建设，重点扶持利民经济技术开发区、宾西经济开发区等6个工业园区。突出地方特色，以建设产业化龙头企业为重点，培植立县（市）产业。做大做强35个省、市级龙头企业，发展龙头在城市、基地在农村的城乡互动型产业。鼓励外资、城市工商资本和民间资本进入农业和农村，扩大城乡经济联合。

　　促进农村劳动力转移。改善农民进城就业环境，清理歧视性规定和不合理收费，依法保障进城就业农民各项权益。推进户籍制度改革，放宽农民进城就业和定居条件。加快小城镇建设，完善城镇载体功能，增强小城镇吸纳农村劳动力的能力。推进12个小康示范镇建设，形成一批工业带动型、旅游商贸型、资源加工型等各具特色的小城镇群体。

　　继续深化农村改革。巩固农村税费改革成果，进一步减轻农民负担。推动农村土地使用权有序流转和土地适度规模经营。深化粮食流通体制改革，全面放开粮食市场，建立对农民的直接补贴制度。加快农村信用社改革，扩大县域企业贷款担保范围，推广农民小额信贷。发展农村新型合作经济组织和市场中介组织，提高农民进入市场的组织化程度。

　　（三）加快发展现代服务业，优化第三产业结构

　　做大做强旅游业。围绕建设世界冰雪旅游名城，大力发展冰雪旅游，打造国际品牌，带动冰雪产业发展。办好第27届哈尔滨之夏音乐会，放大“哈夏”品牌效应，繁荣夏季旅游。整合旅游资源，搞好旅游综合开发，重点发展欧陆风情游、生态游、工业游等产品。加强区域合作，拓展省内旅游线路，联合北方城市及俄罗斯共享旅游资源，构建大旅游格局。继续搞好市场化运作，推进太阳岛风景名胜区、冰雪大世界四季乐园、北方森林动物园、天恒山风景区等10个重点项目建设。加强旅游产品促销。整顿、规范旅游市场，提高从业人员素质。

　　拓展服务业发展空间。继续引进和发展新型商业业态，形成多种经济成分、多层次、多业态并存的商业竞争格局。发展现代物流业，推进哈东物流作业基地、哈西交通运输市场、开发区仓储基地等项目建设。加快国有商业企业改革，启动物资、外贸等行业国有企业改革。大力发展文化产业。发展和规范社区服务业。发展假日经济、会展经济。发展和规范产权交易、会计、审计、评估、法律、物业管理等服务业。

　　加强财税金融工作。搞好财源建设，培育财政收入新的增长点。加强税收征管，打击偷税、漏税、骗税等违法行为。深化财政支出制度改革，提高财政资金使用效益，推动公共财政建设。强化财政监管，严格执行“收支两条线”，严肃查处违规行为。加强政府投资管理，规范企业投资行为，防范和化解金融风险。引进域外资本，启动民间资本，拓宽风险投资渠道。营造良好环境，吸引国内外金融机构到我市设立分支机构。抓住国家政策机遇，积极参与合作银行建设。推进创建金融安全区试点工作，发展资本市场，支持符合条件企业上市融资。积极争取发行企业债券，拓宽企业直接融资领域。

　　推进数字化城市建设。以电子政务为牵动，重点建设6个政务应用系统。完善“中国·哈尔滨”政府门户网站功能，扩大公共服务范围，推广网上并联审批。加快企业信息化建设，畅通网上绿色通道，为中小企业提供信息服务。启动社区信息化服务试点工作，推进农业和服务业信息化，发展软件产业、电子商务和远程教育。

　　（四）开拓国际国内市场，全方位扩大开放

　　以对俄科技经贸合作为重点，积极发展对外贸易。扩大对俄经贸合作规模和领域，加强对俄科技合作重点项目攻关。调整进出口商品结构，高起点引进技术和设备，提高出口产品技术含量。实施名牌战略，培育出口商品知名品牌。鼓励支持区、县（市）和非国有企业发展对外经济贸易，扩大绿色食品和高附加值农产品出口。实施“走出去”战略，鼓励和支持企业跨国生产和经营。发挥劳动力资源优势，进一步开展国际工程承包和劳务输出。加快口岸建设，提高通关效率和服务水平。高质量承办第15届中国·哈尔滨经济贸易洽谈会。

　　加大招商引资力度。把今年作为招商引资年，营造大招商、招大商氛围，实现利用外资新突破。以老工业基地调整改造项目为重点，确定一批招商引资大项目，搞好产业对接，吸引跨国公司战略投资。做好签约项目落实和落户项目服务工作。支持开发区二次创业，强化簇群经济集聚地、高新技术发展高地和招商引资窗口功能，使之成为经济高速增长极、外向型经济先导区。

　　面向国内发达地区，推进横向经济联合。借助沿海发达地区关注老工业基地振兴的机遇，加强横向交流与合作，对经济扩张明显的重点地区和企业实行跟踪招商。发挥省会城市“龙头”作用，加强与省内各地合作。引入国内跨地区资金增长10%。

　　（五）营造全民创业氛围，促进民营经济加快发展把民营经济作为振兴老工业基地的重要力量，消除制约民营经济发展的体制性障碍，支持民营经济向一、二、三产业全面进军。完善相关政策，鼓励民营企业以控股、收购、租赁、联合等形式，参与国有企业改革。出台政策法规，加快发展中小企业。建立健全服务体系，使民营企业在融资、信用担保、法律咨询、技术引进、人才培训等方面享受优质服务。完善协调服务机制，帮助民营企业解决生产经营中的困难和问题。倡导全民创业，建立民间资金投资机制，引导民间资金转化为民间资本。实施民营企业100强发展战略，重点培育和扶持45户科技型、外向型企业。继续实施“小巨人”发展计划，培育和发展一批有优势、有特色、有规模的民营科技企业。

　　（六）以开发人才资源为重点，深入实施“科教兴市”战略

　　坚定不移地走人才强市之路。全面落实《中共中央、国务院关于进一步加强人才工作的决定》，牢固树立人才是第一资源观念，创新人才工作机制，培养、吸引和用好人才，努力营造尊重知识、尊重人才、鼓励创业的社会氛围。抓好高素质专业技术人员队伍培养，高层次人才资源开发，国外智力引进等工作，发展壮大企业博士后工作站。完善吸引人才、留住人才的各项政策。培育和发展人才市场体系，加快市场化配置人才进程。

　　加快区域科技创新体系建设。激活创新源头，推进产学研结合。强化企业创新主体地位，加强国家、省、市级工程技术研究开发中心和技术中心建设。探索社会化、市场化、多元化建设孵化器的新模式，支持哈工大创办工业研究院，支持大专院校和科研院所创办各类科技成果转化中心。加强科技创新服务中心建设，建立和完善生产力促进中心、知识产权交易中心和科技咨询评估中心等中介组织。出台《哈尔滨市专利管理办法》，鼓励以发明专利为目标的科学研究与开发。加大高新技术产业化项目推进力度，启动振兴老工业基地科技行动计划，与清华创业投资公司共同筹集1亿元，建立开放式老工业基地调整改造基金。重点实施大型铝合金型材、联合循环机组等10个重大科研攻关项目。

　　完善国民教育体系。实施“教育发展工程”，深化教育管理和办学体制改革。强化基础教育，完善职业教育，发展高等教育，推进网络教育。建立新机制，增强办学活力，逐步形成公办、民办、社会力量办学共同发展的格局。完善义务教育管理体制，管理使用好转移支付资金。筹集义务教育助学资金1000万元，资助贫困学生5万人。加大特殊教育专项投入，保证困难群体子女、进城务工人员子女和残疾学生接受优质教育。发展职业技术教育，培养应用技术型人才。改造30所中小学危房。

　　（七）突出城市特色和品位，加强城市建设与管理

　　进一步完善城市功能，延展文化内涵。调整行政区划布局，扩大城区面积，进一步拓展未来发展空间。推进松北、哈西、太平、群力等区域性整体规划和开发改造，重点实施与北京中关村开发建设公司、上海世茂集团合作开发松北项目。启动轨道交通一期等大项目，继续实施一批路桥改造工程。加大集中供热力度，新增700万平方米供热能力，城市集中供热普及率达到52%。全力推进磨盘山水库供水工程，完成一期工程总量的60%。改造道里、道外等城区老化供水管网20公里。进一步改善人居环境，实施何家沟整治、沿江污水截流及处理、太平污水处理厂、文昌污水处理厂三期、向阳生活垃圾处理场等项目。继续实施大绿化工程，力争城区人均公共绿地面积达到7平方米。加大环境综合整治力度，继续治理一批污染源，改造城区20条三、四类街路和50条土路，整治街路容貌和出城口地区环境，安装50条无照明街路路灯，建设人行过街天桥5座。加强城市特色景观建设，实施中央大街二期续建、索菲亚教堂二期、沿江风景长廊三期等工程。完善住房保障体系和廉租住房制度，城区拆除成片危棚房120万平方米，改善3万户居民住房条件，解决廉租户住房400户。加强房地产市场宏观调控，规范投资行为。加大清理建设领域拖欠工程款和农民工工资力度，规范建筑市场秩序。

　　切实加强城市管理。完善“两级政府、三级管理、四级网络”管理模式。强化综合执法工作，提高依法管理水平。把环境综合整治向贴近群众生活领域延伸，加快改造旧城区，加强市容环境卫生管理，加大城市交通综合整治力度；调整、延伸公交线路10条，新增、更新公交车辆350台；新增公厕50座。

　　继续搞好经营城市工作。放开经营性和准经营性城建项目，鼓励社会资本参与城市建设和经营。发挥城建投资公司作用，加快建立城建一级融资平台。完善土地收购储备和挂牌拍卖制度，实现土地效益最大化。采取BOT、ABS、合资合作等方式，吸引社会资本参与城建大项目建设。推进城市建设体制改革，实行政企分离。对政府投资建成的公共设施冠名权、广告权，实行统一管理和公开招标拍卖。

　　（八）转变政府职能，改善发展环境

　　积极落实省委、省政府关于开展“招商引资和改善环境年”活动的部署，力争在优化发展环境上实现新突破。

　　以提高行政效能为核心，推进政府管理创新和职能转变。在继续做好经济调节、市场监管的同时，强化社会管理和公共服务职能，把更多的力量放在发展社会事业和改善人民生活上。建立健全突发事件应急机制，提高政府应对公共危机能力。深化行政审批制度改革，减少审批环节，提高审批效率，改进政府管理经济活动方式，真正把经济管理职能转到主要为市场主体服务和创造良好发展环境上来。推行开放式办公，加快建设市行政服务大厅，尽快投入运行。完善公示制、承诺制、首问责任制和失职追究制。加强政务督察，确保政令畅通。建立健全评议和监督政府部门机制，定期听取投资者对政府工作的意见和建议。增强服务意识，帮助企业解决生产经营中的困难。

　　努力降低商务成本。千方百计降低外来投资者的生产经营性费用，严格控制行政事业性和服务性收费，取消不合理收费。全面实行收费许可证制度和企业负担登记卡制度，逐步把我市建成一个商务机遇多、交易成本低、市场秩序好、具有吸引力的城市。

　　维护经营者和投资者合法权益。整顿和规范市场经济秩序，重点完善行政执法、行业自律、舆论监督、群众参与相结合的市场监督体系，严厉打击制假售假、商业欺诈等违法行为，营造公平、公正的竞争环境。围绕政府诚信、商务诚信和社会诚信建设，推动全社会信用体系的建立和完善，打造“信用哈尔滨”。

　　（九）大力促进就业和再就业，完善社会保障体系

　　把扩大就业放在经济社会发展更加突出的位置，走“以人为本”的新型工业化道路，在实施振兴老工业基地战略中，努力培育新的就业增长点，创造更多就业机会，全年安置10万人就业和再就业，再就业率达到40％以上。发展劳动密集型产业，开发公共卫生、城市环保、居民医疗保健以及社区、家政服务等就业岗位。落实税收减免、小额贷款、社保补贴等再就业扶持政策，支持下岗失业人员自谋职业和自主创业。加强劳动力市场建设，强化再就业和创业培训。继续开展再就业援助行动，为困难群体就业提供帮助。继续推行劳动预备制度，延缓就业压力。认真做好大中专毕业生和退伍复员军人的就业安置工作。做好完善城镇社会保障体系试点工作。调整养老保险金计发管理办法，做实养老保险金个人账户，确保国有企业下岗职工基本生活费、企业离退休人员基本养老金按时足额发放，企业基本养老保险费征缴率达到90％以上。推进国有企业下岗职工基本生活保障向失业保险并轨。加强就业、失业登记管理，建立就业保障型的失业保险制度，失业保险参保人数保持180万人。完善城镇职工医疗保险制度，力争参保人员达到140万人。完善城镇居民最低生活保障制度，构筑社会救助体系，把符合条件的城镇困难群众全部纳入城市居民最低生活保障范围，城市居民最低生活保障资金及时足额拨付到位。建立农村特困群众救助制度，救助特困群众10.3万人。建立财政、税收、国有资产变现、社会筹集等多渠道的筹资机制，扩大社会保障金来源。

　　（十）加强民主、法制和精神文明建设，促进各项社会事业协调发展大力发展社会主义民主。贯彻人大及其常委会决议，完善工作报告制度，自觉接受人大监督。加强与政协的联系，自觉接受民主监督。进一步完善与群众利益密切相关的重大事项社会公示制度和听证制度。支持工、青、妇等群团组织开展工作。大力推行政务、村务、企务公开。完善村民自治，加强社区建设，推进社区自治，扩大基层民主。

　　提高依法治市水平。贯彻实施《行政许可法》，建立健全相关制度，明确监督检查职责，逐步建立完善行政执法长效监督机制。加强执法人员专业培训，提高依法行政能力和水平。做好行政复议工作。完善律师、公证、仲裁等法律服务体系。深入开展“四五”普法宣传教育，增强市民法制观念。完善区、县（市）法律援助机构，及时为经济困难群众提供法律咨询。

　　正确处理改革、发展、稳定的关系，切实维护社会稳定。创建“平安哈尔滨”，全力维护社会治安、政治稳定和国家安全。巩固警民打防成果，依法打击各种刑事、经济犯罪，严厉打击“法轮功“等邪教组织和敌对势力的非法活动，坚决扫除黄、赌、毒等社会丑恶现象。加强安全生产管理，落实安全责任追究制度，消除各类安全隐患，防止重特大事故发生。做好信访工作，坚持“及时排查，各负其责，工作在前，预防为主”原则，妥善处置人民内部矛盾引发的群体性事件。

　　推进精神文明建设。开展文明城市、文明村镇、文明行业、文明单位创建活动和军警民共建共育活动，把群众性精神文明创建活动引向深入，提高市民道德水准。深入开展拥军优属工作，巩固全国“双拥模范城”成果。

　　发展各项社会事业。稳步推进事业单位改革。建立健全疾病预防控制体系和医疗救治体系，完成市级和12县（市）疾病控制中心建设，新建市儿童医院综合楼。完善城市社区卫生服务网络。深化农村卫生改革，扩大新型合作医疗试点范围，完成193个乡镇卫生院设备配备和危房改造。建立完善以禽流感为重点的动物疫病防治体系。落实计划生育基本国策，稳定低生育水平。开展全民健身活动，加快市游泳馆等重点体育工程建设。繁荣文化艺术创作、新闻出版和广播电视事业。全面落实民族政策，发展民族团结进步事业。依法加强宗教管理。认真做好外事侨务、对台、残联、老龄、武装、人防、档案和地方志编撰等工作。

　　（十一）求真务实，改进作风，进一步加强政府自身建设

　　按照十六大报告要求，进一步提高各级干部科学判断形势的能力，驾驭市场经济的能力，应对复杂局面的能力，依法行政的能力和总揽全局的能力。深入创建学习型、调研型、决策型、服务型和网络型政府，继续加强“两风”建设，努力把政府工作提高到新水平。

　　推进民主、科学决策。继续实行课题制，逐步完善公众参与、专家论证、政府决策相结合的决策机制。

　　努力解决好人民群众生产、生活中的困难和问题。坚持以人为本，把人民群众的根本利益作为政府工作的出发点和落脚点。深入基层，深入群众，察实情，解难题。做好困难群众救助工作，深入开展“慈善济困民德工程”、“一帮一”、“送温暖”活动。抓好灾民救助和灾区重建工作。落实省委提出的“10项利民行动”，办好我市关系人民群众切身利益的20件实事。

　　大力弘扬求真务实的工作作风。坚持说实话、办实事、求实效，以实为本，实干兴业，把加快发展建立在扎扎实实工作的基础上。坚持从群众中来，到群众中去的工作方法，把群众需要作为第一选择，杜绝形式主义、官僚主义和奢侈浪费行为，制止“形象工程”、“政绩工程”。

　　加强廉政建设。坚持标本兼治、惩防并举，完善监督制约机制，市政府组成人员尤其是我本人，要带头自觉接受人大代表、政协委员、社会各界和广大群众的监督，把党风廉政建设的各项措施落到实处。继续实行廉政责任制，加大查办违法违纪案件的力度，力求从源头上预防和消除腐败。按照“为民、务实、清廉”的要求，着力解决损害群众利益的突出问题，严肃查处各种违纪违法案件，坚决处理征用土地中侵害农民利益，城镇拆迁中侵害居民利益，企业改制中侵害职工合法权益，拖欠和克扣农民工工资等问题。

　　各位代表，振兴老工业基地，全面建设小康社会，是历史赋予我们的使命。让我们紧密团结在以胡锦涛同志为总书记的党中央周围，在省委、省政府和市委的领导下，励精图治，扎实工作，夺取振兴老工业基地和全面建设小康社会的新胜利！
